# Child Abuse, Misdiagnosed by an Expertise Center: Part I—Medico-Social Aspects

**DOI:** 10.3390/children10060963

**Published:** 2023-05-29

**Authors:** Marianne Vlaming, Pieter J. J. Sauer, Emile P. F. Janssen, Peter J. van Koppen, Cornelis M. A. Bruijninckx, Marga W. M. Akkerman-Zaalberg van Zelst, H. A. Martino Neumann, Martin J. C. van Gemert

**Affiliations:** 1 Private Practice, Criminal Psychology and Law, 6986 CL Angerlo, The Netherlands; 2 Department of Pediatrics, Beatrix Children’s Hospital, University Medical Center, 9713 GZ Groningen, The Netherlands; 3 Private Practice, Rehabilitation Medicine & Consultancy, 6137 CL Sittard, The Netherlands; 4 Department of Criminal Law and Criminology, Faculty of Law, VU University Amsterdam, 1081 HV Amsterdam, The Netherlands; 5 Private Practice, 2511 CG The Hague, The Netherlands; 6 Instituut Marga Akkerman, 2101 AD Heemstede, The Netherlands; 7 ZBC-Multicare, 1217 AB Hilversum, The Netherlands; 8 Department of Biomedical Engineering & Physics, Amsterdam University Medical Centers, Location AMC, University of Amsterdam, 1105 AZ Amsterdam, The Netherlands

**Keywords:** infant bruises and rib fractures, Dutch expertise center for child abuse, diagnostic error, misdiagnosed child abuse, medical and social aspects, foster care placement, hypermobility-type Ehlers-Danlos-Syndrome (hEDS)

## Abstract

Child abuse is a dangerous situation for an infant. Professionals need to weigh the risk of failing to act when children are seriously harmed against the serious harm done by carrying out safeguarding interventions. In severe cases, foster care might be advisable. The negative effects for the child’s psychosocial development requires that such placement must be based on very solid evidence. Our aim is to identify why Dutch parents whose child may have a medical condition that could mimic symptoms of child abuse have a significant chance of being erroneously convicted and losing custody of their child. As a method, we describe and analyze the following case. An Armenian-Dutch newborn (uncomplicated term vaginal delivery), starting at two weeks after birth, developed small bruises on varying body locations. At two months, a Well-Baby Clinic physician referred the girl to a university hospital, mentioning that there were no reasons to suspect child abuse and that her Armenian grandmother easily bruised as well. However, before consultation by a pediatrician of the hospital-located Expertise Center for Child Abuse, the parents were suspected of child abuse. Based on the expertise center’s protocols, skeletal X-rays were made, which showed three healed, asymptomatic rib fractures, while invalid statistics suggested, incorrectly, a 10–100 times more likely non-accidental than accidental cause of the symptoms (discussed in Part II of this series). The expertise enter physician ignored any argument that could show parental innocence, including the positive parent-child relationship reported by the Well-Baby Clinic and the general practitioner. The girl and her older brother were placed in a family foster home and then in a secret home. The case radically resolved when a large bruise also developed there, and an independent tissue disease specialist diagnosed a hereditary connective tissue disorder in the mother, implying that the girl’s bruises and rib fractures could well be disease-related. In conclusion, if child abuse is suspected, and foster care placement considered, the patient and the parents should be thoroughly investigated by an independent experienced pediatrician together with an experienced pediatric clinical psychologist or psychotherapist to produce an independent opinion. Children deserve this extra safeguard before being separated from their parents.

## 1. Introduction

The American Child Abuse Prevention and Treatment Act defines child abuse and neglect as, at a minimum: “Any recent act or failure to act on the part of a parent or caretaker which results in death, serious physical or emotional harm, sexual abuse or exploitation; or an act or failure to act, which presents an imminent risk of serious harm”. This English phrasing is an excellent translation of the Dutch child abuse definition as has been laid down in legislation. In the Netherlands, child abuse and neglect have an estimated incidence of approximately 1700 cases per year [1]. Child abuse is a condition that can jeopardize the development of an infant or child [2,3]. Parents may lose, due to the abuse, the custody of their child. Children may be placed in foster homes, losing contact with their parents. This may have a long-lasting negative effect on the child’s psycho-social development [4,5,6,7,8,9,10].

Diagnosing a case of child abuse can be very difficult, especially when the parents deny any form of involvement [11,12]. In these cases, extensive medical examinations, preferably by pediatricians specialized in rare disorders or by clinical geneticists, are imperative in order to rule out any other explanation for the signs and symptoms found in the infant. Special attention must be given to rare diseases that may show symptoms similar to those frequently seen in child-abuse cases and there should be a willingness to consider such diseases as an alternative diagnosis [13]. This also includes extensive psychosocial examinations of the child and their family by an experienced clinical psychologist, or a psychotherapist specialized in child development.

Because of the impact and potential consequences of child abuse, several pediatricians in the Netherlands have instituted a national expertise center that could assist pediatricians in how to deal with cases of potential child abuse.

In this paper, Part I of a series of three related papers, our main aim is to identify why Dutch parents whose child may have a medical condition that could mimic symptoms of child abuse have a significant chance of being erroneously convicted of child abuse and losing custody of the child. As a method, we describe and analyze a case where the diagnosis of child abuse was made, supported by the expertise center, but where the symptoms were due to an unrecognized underlying disease. We also wish to express the need for a thorough medical and social assessment in cases where child abuse is suspected. The complexity of the statistical method used, as related to Bayes’ theorem, required a separate Part II [14], and readability required a forthcoming Part III on the legal and moral aspects. However, the highlights of Parts II and III are summarized in this Part I.

### Expertise Center and Child Protection Organizations

Essential roles in this case were played by the Dutch Expertise Center for Child Abuse [14,15]), expressed henceforth as “the expertise center”, and three Dutch Child Protection Agencies.

The expertise center is a foundation which has a unique non-governmental partnership between the pediatric departments of three Dutch university hospitals and the forensic medicine department of the Netherlands Forensic Institute. Their methodology is [15] to be permanently available for healthcare professionals by pediatricians who have had 2.5 years of additional education in child abuse diagnostics. For physicians, they offer easily accessible but anonymous advice without requesting permission from parents or caregivers and without observing the child themselves. Pediatricians are the most prevalent advisees, followed by child protection employees. The advice requesting physician will receive a letter stating the anonymous information provided by him/herself, the conclusions reached by the expertise center and, if needed, the advice for further assessment or follow-up. The foundation is not involved in further treatment or follow-up of the cases. Expertise center physicians work with likelihood ratios, part of Bayes’ theorem, to come to a conclusion. In 2018, they received 229 advisory requests [15].

## 2. Case Report

**Figure 1:**
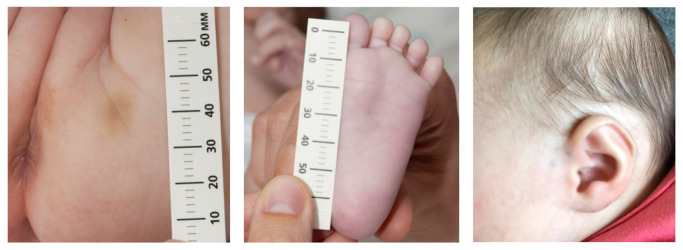
Examples of the small bruises.

An Armenian(father)-Dutch girl (second child, uncomplicated term vaginal birth, 2970 g), was born in 2020. From about two weeks after birth, she developed small bruises on varying body locations, particularly the extremities, foot soles and buttocks. There were never bruises located on her chest. Figure 1 shows some typical examples. The mother consulted the Well-Baby Clinic, where the physician suggested a wait-and-see approach, and to take pictures, which were all openly shared with everybody that became involved in this case, including the general practitioner. After two months, when the infant was still developing bruises, the physician of the Well-Baby Clinic referred the girl to the pediatrics department of a university hospital to investigate whether there was a medical condition that caused these bruises. The referral letter stated that the paternal grandmother easily bruised too and that there were no indications at all for child abuse suspicion. The consultation was performed by a pediatrician who suspected physical child abuse before having seen the patient. The pediatrician missed identifying the infant’s skin hyperextensibility and joint hypermobility. Indications for a connective tissue disorder were considered unlikely. The protocol of the hospital required immediate hospitalization together with her 20-month-old brother. The parents refused admission and denied child abuse. The university-based pediatrician then consulted an expertise center pediatrician for immediate advice. Without having seen the infant, and without having talked with the parents, this pediatrician did not properly investigate the possibility of a genetic disorder and advised the hospital admission of the girl and her 20-month-old brother and, if needed, by order of a judge. Due to the threat of child removal when refusing participation, the parents complied with the hospital’s treatment plan. Protocol-based X-ray skeletal status showed three asymptomatic, healed, not dislocated, antero-lateral rib fractures at the left side. The bruises and rib fractures completed the expertise center pediatrician’s diagnosis of child abuse. This triggered investigation of her brother using the same extensive protocol as the girl. No abnormalities or signs of abuse were found. Based on the bruises and rib fractures, a clinical geneticist (actually a pediatric resident) was consulted who concluded, without available argumentation, that the girl had no signs of a connective tissue disease.

The expertise center was consulted again and concluded, with their protocol-based Bayesian likelihood ratios, that this case had a 10–100 times larger probability of having a non-accidental than an accidental cause. In Part II [14], we show that this conclusion is statistically invalid. Both children were then, by court order, placed in foster care with their grandparents. A child protection physician declared aggravated assault to the police. The next day, the police removed the children into an unknown foster house. Several weeks later, both children were placed at their maternal aunt, albeit still not at their parents’ home. An innocent incident in the foster care family had caused a new bruise of about 4 × 2 cm^2^ on the girl’s left lower leg. The pediatrician of the university hospital did not consider the bruises to be a result of physical abuse by the foster parents, nor consulted the expertise center, and ended their involvement in the case abruptly, reporting that they would investigate again if bruises reappeared; however, there was no consideration of parental innocence.

Then, two events allowed the children to return home. The first was the mother’s diagnosis of hypermobility-type Ehlers-Danlos syndrome (hEDS) and the fact that her children also had signs of the disease (Paragraph 3). The second event was a second opinion by another university hospital, where the assigned pediatrician confirmed that the mother’s hEDS could certainly have affected the girl too, and that her rib fractures could well have been developed perinatally; furthermore, the assigned pediatric radiologist had observed widened metaphyses and concluded that an underlying bone disease could well be possible. The children finally returned to their parents. After eight months, the 4th civil court ordered the end of all child protection measures, again without expressing parental innocence.

The case required five court sessions, four civil and one criminal (see Part III). 

## 3. Hypermobility-Type Ehlers-Danlos Syndrome: Family Evaluation

Ehlers-Danlos syndromes (EDS) are a group of autosomal dominant disorders of connective tissue, part of the spectrum of inherited metabolic diseases, that are classified into 14 types in the way they affect the body and in their genetic causes, e.g., [16,17,18,19,20]. The most prominent features observed in EDS include a triad of joint hypermobility, skin hyper-extensibility and generalized fragility of soft tissues. However, the extend duration and severity of symptoms vary significantly between EDS subtypes [21]. In all but the hypermobile subtype (hEDS), genetic variants have been identified as the cause for the disorder and are part of the diagnostic criteria. Since the publication of the 2017 criteria for EDS (Malfait et al. [16]), other genes have been identified describing additional new types. The EDS International Consortium proposed the 2017 International Classification for the EDS [17] to replace the Villefranche nosology which came out in 1998. In an attempt to better define the disease subtypes and reduce future misdiagnosis, the new criteria for hEDS were more strict than previous criteria [22]. Although the clinical assessment of hEDS is not difficult, examiners should be trained to ensure the reliability of the examination [23]. The incidence of the EDS/Hypermobility Syndrome is not well known, but is reported to be between about 1:5000 [24] and 1:500 [25], see also Malfait et al. [20]. Children have a 50% probability of acquiring the disorder too [20]. Clinical practice, as well as research studies, have demonstrated that 90% of the hEDS cases are female [21].

Characteristic symptoms of hEDS include joint hypermobility and instability, recurrent joint subluxations or dislocations, chronic widespread joint pain, skin manifestations, and a wide variety of associated symptoms and comorbidities, including migraine headaches, functional bowel disorders, orthostatic intolerance, and chronic fatigue. Symptoms of hEDS in the pediatric and adolescent population can range from minimal effects to significant pain, fatigue and medical complications leading to disability [26]. Joint hypermobility is a common, though largely ignored, physical trait with increasing clinical reverberations [27]. Joint hypermobility is characterized by an extended range of motion of joints, increased distensibility of joints in passive movements and hypermobility in active movement in the absence of other rheumatologic disease [23]. Feeding difficulties due to functional gastrointestinal (GI) symptoms (i.e., nausea, pain, and bloating) are also well described in patients with hEDS [28,29].

Establishing the diagnosis of EDS/hEDS is often problematic for patients. Initial symptoms and EDS-associated diagnoses can appear to be simply a ‘normal’ pattern of childhood illness when taken as an isolated event. A mean of 14 years elapses between the first clinical manifestations and the actual diagnosis [21]. There is a large gender difference in the age of diagnosis. Scicluna et al. [21] report that the age of first diagnosis peaks in the age group of 5–9 years for men and 15–19 years for women. For men, 72% were diagnosed during childhood (age < 18 years) compared to only 41% for women. For 25% of patients, the delay lasted over 28 years. A misdiagnosis was given to 56% of the patients resulting in inappropriate treatment in 70% of these patients [21].

Management guidelines for adults with hEDS focus on symptomatic treatment with physical therapy and other interventions, with the goal to improve daily function and to prevent deconditioning. However, there is no consensus for treatment of hEDS manifestations in the pediatric population, or which aspects of management optimize the quality of life. Management of children and adolescents with hEDS can be challenging due to chronic pain and multisystem complaints, and often requires multidisciplinary care [26,29].

The clinical diagnosis of hEDS is based on the simultaneous presence of symptoms belonging to three different categories (Table 2 of [22]). Criterion 1 includes generalized joint hypermobility, to be assessed by the Beighton score with at least six out of nine for children and adolescents, five out of nine for adults up to 50 years of age, and four out of nine in adults over 50 [22]. The Beighton score [30] is a test that measures joint hypermobility (flexibility). It involves simple maneuvers, such as bending your pinkie (little) finger backward to check the joint angle. Please see the video demonstration of the test in [30]. The Beighton score uses a nine-point scoring system. The higher your score, the more flexible your joints are [30]. Limitations such as prior surgery or joint injury should be taken into consideration, as well as a history of hypermobility; a five-point questionnaire was developed for this purpose (Table 3 of [22]). A ‘yes’ answer to two or more of these questions would suggest joint hypermobility and add one additional point to the Beighton score. For criterion 2, patients must meet two or more of the following: systematic manifestations of a more generalized connective tissue disorder, positive family history and/or musculoskeletal complications. Criterion 3 requires all of the following: absence of unusual skin fragility that would prompt consideration of other types of EDS, exclusion of other heritable and acquired connective tissue disorders and exclusion of alternative diagnoses that may also include joint hypermobility [22].

Furthermore, EDS is poorly recognized in children, from lack of awareness by physicians and child protection personnel, frequently causing misdiagnosed child abuse [21,31,32,33,34,35,36,37,38,39] with temporary or even permanent foster care placement.

In this case, the whole family was evaluated for the diagnosis hEDS (EPFJ) using the diagnostic criteria for hEDS (Malfait et al. [16]). The maternal grandmother did not meet the diagnostic criteria for hEDS. The medical history of the maternal grandfather suggested that he might be symptomatic of a connective tissue disease. The mother fully met the diagnostic criteria for hEDS. Because the diagnostic criteria lack reliability in young children, the child could not be evaluated. She did have a Beighton score of 4/8 on physical examination, which is considered normal. Because of her young age and the shortcomings of the diagnostic criteria for hEDS, this was not conclusive. Her brother did show generalized hypermobility and also had several other signs suggesting a diagnosis of a connective tissue disease/hEDS.

## 4. Discussion

Whenever possible, children should be raised by their own parents. Only in exceptional cases, e.g., severe physical child abuse, might foster care be preferable. Given the high risk of a negative effect of placing a child in a foster home, such a decision must be based on very solid grounds [4,5,6,7,8,9,10,40]. The family, as well as each caretaker, needs to be carefully interviewed individually. Information about attitudes and expectations in the family towards the child(ren), discipline, work and leisure activities should be gathered. Particular attention needs to be given to the history of each parent’s own childhood and whether or not they have been abused themselves, see [31] and part III of this series. A thorough review of individual, parental, familial, environmental and social network characteristics should be part of the procedure too [41]. In this case, the hospital reported suspected abuse to the child protection officials, whose opinion was based on an incomplete medical history and physical examination, inadequate family assessment and invalid use of Bayesian statistics (discussed in Part II of this series). The child protection agencies followed the reports made by the expertise center pediatricians without comment and, consequently, judges also accepted all of their conclusions. Why were the parents, despite being innocent, accused of child abuse?

The misunderstanding started with the physician who performed the first examination. The fact that the pre-mobile infant had bruises made this pediatrician suspect a case of child abuse beforehand. However, Bilson described the following reasons for accidental bruises in children who were not yet able to roll over: bumping into the mother’s tooth, falling asleep on a dummy, banging themselves with a fist or rattle, or a toy that was dropped on the baby [42,43]. In this case, the pediatrician ignored a range of similar explanations offered by the parents and grandparents of the child. That the symptoms started only two weeks after birth, that the referring Well-Baby Clinic physician wrote that there were no reasons at all to suspect child abuse, and that the paternal grandmother was also easily bruised made no difference for this physician. Inadequate examination continued with two other important consultations: the girl by a clinical geneticist, and her brother by a pediatrician. However, both were pediatric residents. The expertise center was consulted when the parents refused the admission of both of their children to the hospital. Their pediatrician concluded, based only on the digitally sent pictures and without having talked with the parents, that this case was highly suspicious for child abuse. When the X-rays showed three rib fractures, it was clear to that pediatrician that this was a case of severe physical abuse. Placing the children immediately in foster care was indicated. Further investigations on whether there might be an underlying disease, causing both the fractures and the bruises, were only partly performed.

Despite the work by Bilson [42,43], other studies have shown that it can be difficult to find a medical cause for bruising in very young infants, e.g., [44,45,46,47]. This, however, should not result in a wrong diagnosis of child abuse. EDS, particularly hEDS, should always be considered in the differential diagnosis of children with a suspicion of non-accidental injury [21,31,32,33,34,35,36,37,38,39]. Extensive personal and family assessment is needed [40], preferably in the setting of a highly specialized center. The fact that both the girl and her paternal grandmother were easily bruised should have motivated the pediatricians to further investigate the family history. They would then have learned that the mother easily bruised as well, that she had an unusually soft, doughy skin, mild skin hyperextensibility, easily forming temporomandibular joint luxation, piezogenic pedal papsules and that she had remaining scars from the bariatric surgery she had undergone years earlier. All these facts were suggestive of a possible genetic tissue disorder. A 2015 issue of the *American Journal of Medical Genetics C* (Seminars in Medical Genetics) [17], was devoted to the role of a medical geneticist or pediatrician with special knowledge of genetics and rare diseases to differentiate child abuse from rare diseases. In that issue, e.g., Shur and Carey [48], as well as in the paper of Christian and Slates [13], special attention was given to EDS. It is surprising therefore that the parents were not investigated to rule out EDS as a potential cause of the girl’s symptoms. The patient herself was too young to diagnose or rule out this disease. However, the bruise that also developed in the secret foster home strongly suggested EDS-inheritance of the girl.

Expertise center physicians considered the ribs fractures an additional proof of child abuse despite the fact that these fractures are virtually always asymptomatic, as in this case, and thus of unknown incidence [49]. Infant rib fractures may indicate child abuse but they do not prove it. Rib fractures in newborns may be due to other causes, such as prematurity and birth trauma [50]. Furthermore, Högberg and Thiblin [49] found in their case series that “rib fractures in young infants, diagnosed as abuse, are usually asymptomatic and healing (as in the present case). A substantial proportion had metabolic risk factors, suggesting false positive cases”. Risk factors for rib fractures are maternal overweight and vitamin-D deficiency [49,51], both present here. Moreover, rib fractures due to child abuse have been reported as mainly posterior fractures, e.g., Figure 2 of [52], while they were antero-lateral here. In Part II [14], we argue from the literature that children of hEDS mothers develop bone fragility, most severe in hEDS children, which is highly likely in our case. We therefore hypothesized that the girl’s rib fractures developed during delivery, as birth trauma due to her weak bones.

The expertise center pediatricians’ third indicator for child abuse was the large likelihood ratio estimation of 10–100, which was based on data from the literature on bruises and rib fractures. However, we show in Part II [14] that likelihood ratios only express how much more frequently symptoms develop when abuse actually did occur, compared to a non-abuse cause. It is therefore obvious that a likelihood ratio does not provide the probability that the observed symptoms were caused by abuse. Bayesian statistics include the abuse incidence in the abuse versus non-abuse probability of the symptoms as the product

Probability (abuse/non-abuse) = (likelihood ratio) × (abuse incidence)(1)

In words:

Relative probability that the symptoms are due to abuse =

Relative probability of the symptoms if abuse did occur × Abuse incidence

For young infants, the physical abuse incidence is obviously very small, estimated from the literature as ≈0.002 [14], implying that correct use of Bayes’ theorem predicts, virtually always, that symptoms have a negligible probability of abuse versus non-abuse. Importantly therefore, a patient should never be diagnosed on the basis of (Bayesian) statistics.

A National Expertise Center for Child Abuse can have an important role in making both physicians and society aware of the dangers of child abuse. It might also help local doctors to analyze a case of potential child abuse. The problems start when these physicians make reports and draw conclusions without seeing the patient and without talking with the parents. A wrong diagnosis of child abuse can also be expected when expertise center physicians have the attitude that when there is high suspicion of child abuse, abuse must be the cause of symptoms, unless another explanation might be found. The expertise center pediatricians demonstrated a lack of knowledge of rare diseases in which the symptoms might mimic a case of child abuse. Furthermore, they misused Bayes’ theorem by taking likelihood ratios equal to the probability that abuse caused the symptoms rather than using Equation (1), thus predicting orders of magnitude larger abuse probabilities than obtained from Bayes’ theorem (Part II [14]). In our opinion, therefore, these physicians should have further education in recognizing rare diseases of which the signs and symptoms mimic those often seen in child abuse cases, in Bayesian statistics, and particularly in the clinical importance of seeing the child and talking with—and listening to—the caregivers.

Finally, Bayes’ theorem is made up of complex statistics, and it is likely that physicians, child protection workers and judges have, at best, incomplete understanding of what it is and does. For parents, it is almost impossible to fight against the opinion of well-respected experts from a university hospital and the expertise center for child abuse, despite the fact that they may be abuse-fixated and not be experts in rare diseases or Bayesian statistics.

In conclusion, when a case of suspected child abuse occurs, and foster care placement of the child is considered, we suggest that an independent pediatrician with a wide experience in pediatrics, in close corporation with a clinical psychologist or a psychotherapist specialized in the development of children, examine the patient, do investigations of the parents and produce an independent opinion. Concerns within health or social care about the child and family should always be assessed in a multidisciplinary way. The seriousness, complexity and prevalence of child abuse require high standards for the quality of child safety procedures. The best interest of the child should form the basis of every child protection investigation. This examination not only needs to focus on the child’s physical and psychological safety and its development (discussed in Part III of this series) but also on building relationships rather than developing suspicion [42]. Children, their parents and other family members deserve this extra safeguard.

To prevent cases such as this from occurring again therefore requires that expertise center physicians stop using Bayesian statistics, and certainly invalid likelihood ratios, stop using their anonymous methodology and increase their knowledge of rare and metabolic diseases that may present as symptoms that could mimic child abuse. The parents should be thoroughly investigated by an independent experienced pediatrician together with an experienced pediatric clinical psychologist or psychotherapist to produce an independent opinion. Children deserve this extra safeguard before being separated from their parents.
